# Effects of Immunosuppressive Drugs on Viability and Susceptibility of Adipose- and Bone Marrow-Derived Mesenchymal Stem Cells

**DOI:** 10.3389/fimmu.2015.00131

**Published:** 2015-04-16

**Authors:** Wakako Tsuji, Jonas T. Schnider, Meghan M. McLaughlin, Riccardo Schweizer, Wensheng Zhang, Mario G. Solari, J. Peter Rubin, Kacey G. Marra, Jan A. Plock, Vijay S. Gorantla

**Affiliations:** ^1^Department of Plastic Surgery, University of Pittsburgh, Pittsburgh, PA, USA; ^2^Department of Surgery, Shiga Medical Center for Adults, Moriyama, Japan; ^3^Department of Plastic Surgery and Hand Surgery, University Hospital Zurich, Zurich, Switzerland; ^4^McGowan Institute for Regenerative Medicine, University of Pittsburgh, Pittsburgh, PA, USA; ^5^Department of Bioengineering, University of Pittsburgh, Pittsburgh, PA, USA

**Keywords:** adipose-derived stem cells, anti-lymphocyte serum, bone marrow-derived stem cells, cell size, immunomodulation, tacrolimus, viability, susceptibility

## Abstract

The immunomodulatory potential of cell therapies using adipose-derived stem cells (ASCs) and bone marrow-derived mesenchymal stem cells (BM-MSCs) has been studied in vascularized composite allotransplantation (VCA). Most cell therapy-based experimental and clinical protocols integrate some degree of recipient conditioning/induction with antibodies or other immunosuppressive agents. We investigated the susceptibility of ASCs and BM-MSCs to anti-lymphocyte serum (ALS) and tacrolimus. Rat ASCs and BM-MSCs were exposed to varying concentrations of tacrolimus and ALS *in vitro*. Serum from ALS-treated animals was added to cell cultures. Viability, susceptibility, and cytotoxicity parameters were evaluated. ALS inhibited ASC and BM-MSC viability and susceptibility *in vitro* in a dose-dependent manner. ASCs were more susceptible to both ALS and tacrolimus than BM-MSCs. Trypsinized and adherent ASCs were significantly smaller than BM-MSCs. This is the first report on the viability and susceptibility characteristics of BM-MSCs or ASCs to collateral effects of ALS and tacrolimus. These *in vitro* insights may impact choice of cell type as well as concomitant conditioning agents and the logistical coordination of the timing, dosing, and frequency of drug or cell therapy in solid organ transplantation or VCA protocols.

## Introduction

Vascularized composite allotransplantation (VCA) is an emerging clinical realm of solid organ transplantation (SOT), that deals with the restoration of devastating tissue defects secondary to trauma, congenital malformations, or oncological surgery. VCAs such as face, limb, or abdominal wall transplants involve multiple tissues such as skin, fat tissue, muscle, cartilage, tendon, bone, bone marrow, lymph nodes, nerve, and vessels among others, which create a heterogeneous and highly complex immunogenic environment. In the past 15 years, over 100 upper extremity and 30 face transplantations have been performed under conventional immunosuppression protocols similar to SOT with encouraging functional, immunologic, quality of life and graft survival outcomes (1). One key barrier for routine clinical feasibility of such life-enhancing VCA in the need for prolonged immunosuppression with its coincident adverse effects.

Mesenchymal stem cells (MSCs) represent heterogeneous cell populations present in multiple tissues (2). Adipose-derived stem cells (ASCs) and bone marrow-derived mesenchymal stem cells (BM-MSCs) can differentiate along multiple mesenchymal lineages, such as adipocytes, osteocytes, chondrocytes, myocytes, and Schwann cells. ASCs and BM-MSCs have been studied for their tissue repair properties (3, 4). Some soluble factors are constitutively secreted by MSCs, whereas others are induced when MSCs are exposed to appropriate inflammatory environments (5). These factors can promote angiogenesis, tissue regeneration, extracellular matrix remodeling, cellular recruitment, and immune cell activation or suppression acting via local or paracrine mechanisms. Multiple precursor cells, endothelial cells, and pericytes are components of MSCs (6). These cells respond and home to sites of injury (7).

Mesenchymal stem cells also possess immunomodulatory and immunosuppressive properties (5, 8) and experimental studies have demonstrated that MSCs prolong cardiac and skin graft survival (9–11). Thus, cell therapies utilizing allogeneic ASCs or BM-MSCs can expand the scope, relevance, and impact of their immunomodulatory applications in VCA as well as SOT.

Bone marrow-derived mesenchymal stem cells have been widely studied in clinical applications (12–14); however, studies using ASCs in either VCA or SOT are limited. MSCs have been evaluated in clinical protocols to support engraftment in hematological stem cell transplantation, yet some experimental limitations remain. Paracrine function seems to contribute to immunomodulation, but the mechanistic basis remains unclear. MSCs have a propensity for entrapment in the lung capillaries and liver sinusoids. Various strategies to overcome these limitations, including repetitive infusion of MSCs (11), preparatory vasodilation, and peripheral arterial administration have been suggested (15). ASCs seemingly have advantages over BM-MSCs in terms of clinical translation as cell therapies. One gram of aspirated adipose tissue yields approximately 3.5 × 10^5^–1 × 10^6^ ASCs compared to 5 × 10^2^ − 5 × 10^4^ BM-MSCs isolated from 1 g of bone marrow aspirate (16). Higher cell yields, ease of procurement, expeditious processing, and reportedly superior immunomodulatory characteristics support use of ASCs in cell therapies (17).

Induction therapy with polyclonal [anti-lymphocyte serum (ALS) or anti-thymocyte globulin (ATG)] or lytic monoclonal antibodies alemtuzumab (Campath^®^ 1H) for lymphocyte depletion followed by maintenance treatment with a calcineurin inhibitor (tacrolimus) has been used in both experimental and clinical SOT or VCA. Most stem cell-based therapies currently in Phase I or IIa/b clinical trials in SOT or VCA incorporate recipient conditioning protocols with antibodies/biologics or calcineurin inhibitors. There is thus a need to investigate the susceptibility of stem cells such as ASCs or BM-MSCs to such coincident or simultaneous conditioning treatments. The purpose of this study was to evaluate the collateral effects of ALS or tacrolimus on the viability and susceptibility characteristics of these cells *in vitro*.

## Materials and Methods

### Animals

Six- to eight-week-old male Lewis (RT1^1^, recipient, LEW) and Brown Norway (RT1*^n^*, donor, BN) rats weighing 250–300 g (Harlan, Indianapolis, IN, USA) were maintained in a specific pathogen-free environment at the University of Pittsburgh. Experiments were performed in accordance with University of Pittsburgh Institutional Animal Care and Use Committee (IACUC) guidelines and approved protocols.

### Isolation of rat ASCs

For stem cell isolation, adipose tissue was collected from bilateral inguinal fat pads and epididymes of BN rats. On average, 45 g of fat tissue was harvested from three rats. Enzymatic fat digestion was performed by collagenase type II (Worthington Biochemical Corp, Lakewood, NJ, USA) and bovine serum albumin (Millipore, Billerica, MA, USA) in Hanks’ balanced saline solution (Cellgro Mediatech Inc., Manassas, VA, USA) for 60 min at 37°C. The digested tissue was centrifuged at 1,000 rpm for 10 min. The cellular pellet [stromal vascular fraction (SVF)] was resuspended in erythrocyte lysis buffer and filtered with sterile gauze. SVF was transferred to sterile culture flasks with Dulbecco’s modified Eagle’s medium (DMEM; Cellgro Mediatech, Inc.) plus supplemental Ham’s F-12 medium (Gibco, Grand Island, NY, USA). After overnight incubation for cell attachment, non-adherent cells were removed using a phosphate buffered saline wash. The attached ASCs were cultured in DMEM/F-12 supplemented with 10% fetal bovine serum (ATLAS Biologicals, Fort Collins, CO, USA), 0.1 μM dexamethasone (Sigma-Aldrich, St. Louis, MO, USA), 1% penicillin–streptomycin (Gibco), and 1.25 mg/L amphotericin B (Gibco). SVF from 45 g of fat tissue was plated in a T75 flask (BD Falcon). Approximately, 5 × 10^5^ confluent ASCs were obtained (passage 0). ASCs were expanded *in vitro* until passage 3.

### Isolation of rat BM-MSCs

Bone marrow-derived mesenchymal stem cells were obtained from BN rats by flushing their appendicular bones with Roswell Park Memorial Institute 1640 (RPMI 1640; Lonza, Walkersville, MD, USA). Three rats were used to isolate BM-MSCs. The cellular pellet was resuspended in erythrocyte lysis buffer for 3 min. The reaction was stopped by adding RPMI 1640 supplemented with 10% fetal bovine serum (ATLAS Biologicals), 2.5 μM HEPES (Sigma-Aldrich), 1% penicillin–streptomycin (Gibco), 1.25 mg/L amphotericin B (Gibco), 1% l-glutamine (Gibco), and 1% Sodium pyruvate (Gibco), 1% 2-mercaptoethanol (Gibco; basal media for BM-MSCs), and then centrifuging at 1,750 rpm for 5 min. Cells were resuspended with the basal media for BM-MSCs and plated into cell culture flasks. On average, 5 × 10^5^cells were obtained once BM-MSCs were confluent in a T75 flask (passage 0). BM-MSCs were expanded *in vitro* until passage 3.

### Cell characterization

Third-passaged BN rat ASCs and BM-MSCs were characterized using flow cytometry. Aliquots of 5 × 10^5^ cells for each cell type were stained with anti-rat CD29, CD90, CD45 (eBioscience, San Diego, CA, USA), and CD73 (BD, San Jose, CA, USA) antibodies. The cells were then analyzed using a BD LSRII flow cytometer (Becton Dickinson, Franklin Lakes, NJ, USA). Data were analyzed with FlowJo software (TreeStar Inc., Ashland, OR, USA).

### Imaging and cell characterization with ImageJ

Plates were imaged and cell phenotype was characterized under a Nikon Eclipse TS100 (Nikon Instruments Inc., Melville, NY, USA) at the University of Pittsburgh Center of Biologic Imaging. Cell sizes were calculated with ImageJ software (National Institute of Health, Bethesda, MD, USA).

### Proliferation and viability assays

Cells were suspended in the basal medium, plated in 96-well microplates (BD Falcon™) at a density of 1,000 or 5,000 cells/well, and cultured for 1 and 7 days. MSC and ASC viability was evaluated by MTT assay using a commercially available kit (Millipore, Billerica, MA, USA). Absorbance was measured at 570–630 nm with a plate reader (Infinite M200 PRO, Tecan, Morrisville, NC, USA). The CyQUANT^®^ Cell Proliferation Assay Kit (Invitrogen, Carlsbad, CA, USA) was used to evaluate cell proliferation, utilizing green fluorescent dye that binds to cellular nucleic acids. To assess the influence of ALS and tacrolimus on stem cell viability and proliferation, rabbit anti-rat lymphocyte serum (25 μL; ALS; CEDARLANE^®^, Burlington, NC, USA) at different dilutions (0, 2.5, 5, 10, 20, and 40 μL/mL) was added to the cells, replacing the medium. Alternatively, tacrolimus (LC Laboratories, Woburn, MA, USA) was added at different concentrations (2, 10, 50, 250, and 1250 ng/mL). For the agents, *in vivo* serum concentrations were calculated as 10–20 μL/mL for ALS and 10 ng/mL as trough levels for tacrolimus. In a different secondary setting, serum from ALS-pretreated Lewis rats (on injection days −5 and 0) was used *in vitro* to evaluate the longer-term depletional effects of ALS. BN rat ASCs and BM-MSCs were seeded at the density of 5,000 cells per well on day 1. Blood was collected from naïve (*n* = 3) and ALS-treated rats (*n* = 3) on days 0, 1, 3, and 7. The serum of ALS-treated or naïve rats was added to the cells instead of culture medium. MTT assay was performed in triplicate using serum from each animal after 2 h of incubation.

### Complement-dependent cytotoxicity assay

Complement-dependent cytotoxicity (CDC) assay was performed using aCella™-TOX (Cell Technology, Inc., Mountain View, CA, USA) to simulate transplantation-related complement-associated cytotoxicity. Briefly, 1 day before the experiment, cells were seeded at densities of 5,000 and 10,000 cells/25 μL/well in a 96-well microplate. Rabbit anti-rat lymphocyte serum (25 μL; ALS; CEDARLANE^®^, Burlington, NC, USA) at different dilutions (0, 2.5, 5, 10, 20, and 40 μL/mL) was added. There was no antibody concentration provided by the manufacturer. The plate was shaken for 30 s and then placed in a 5% CO_2_ incubator for 15 min at 37°C to allow the cells to opsonize. Rat complement serum (25 μL; Fitzgerald Industries International, Acton, MA, USA) was added to the appropriate wells to start the reaction. Upon shaking for 30 s, the plate was incubated at 37°C in the CO_2_ incubator for 30 min. The plate was then removed from the incubator and allowed to cool down to room temperature (RT) for 15 min. The target cells in the maximum lysis control wells were then lysed by adding 10 μL of the lysis buffer, and the plate was incubated on the bench top for an additional 5 min at RT. One-hundred twenty five microliters of basal medium was added to each well to reach a total volume of 200 μL. The plates were centrifuged for 1 min at 750 rpm. Fifty microliters of the enzyme assay diluent was transferred to the appropriate wells of an opaque white luminescence plate. Fifty microliters of each reaction supernatant was transferred to wells containing the assay diluent. One-hundred microliters of 2× enzyme assay reagent (containing G3P), followed by 50 μL of 1× detection reagent was added to each diluted supernatant. The plates were again shaken for 30 s and immediately read in luminescence mode using the plate reader at 5 min intervals.

### Statistical analysis

InStat (Version 3.0, GraphPad, San Francisco, CA, USA) was used for statistical analysis. Data are presented as means ± SD. Differences between the groups or sites of measurements were assessed by unpaired analysis of variance. A *P* value of <0.05 was considered statistically significant.

## Results

### Cell characterization, morphology, and size

**Figure 1:**
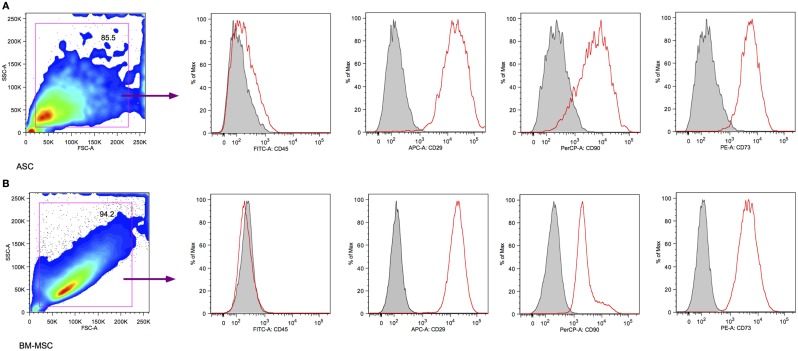
**Surface marker characterization by flow cytometry**. Both third-passaged BN rat ASCs **(A)** and BM-MSCs **(B)** were assessed by flow cytometry and revealed a CD45^−^CD29^+^CD90^+^CD73^+^ surface marker phenotype.

**Figure 2:**
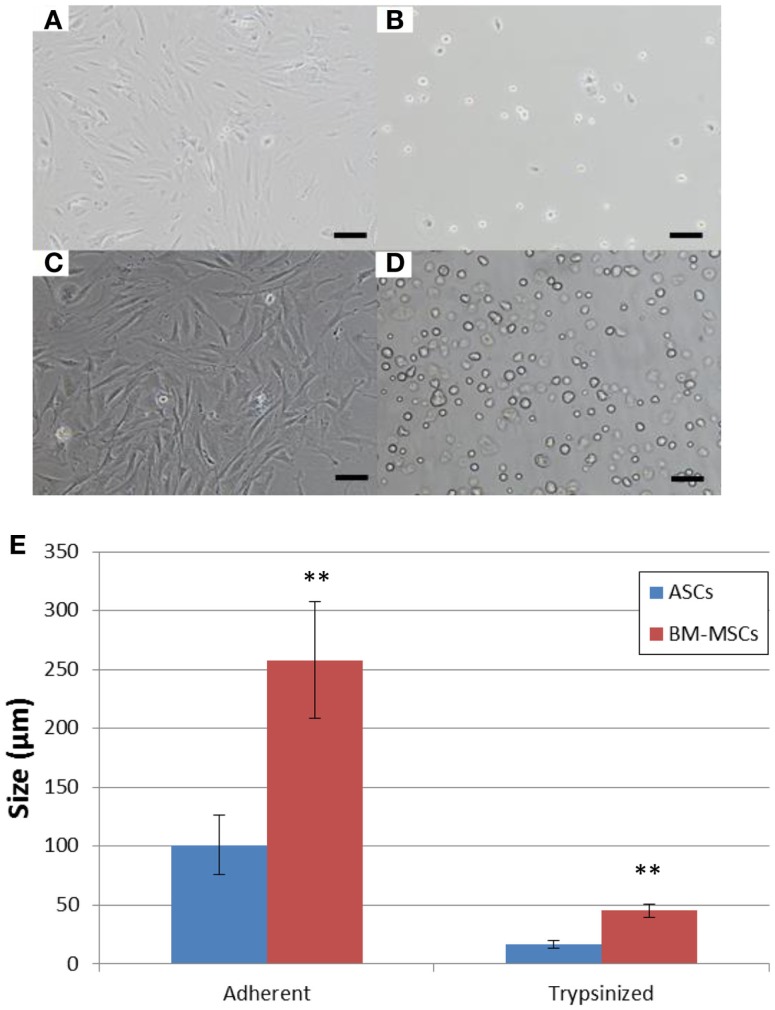
**Cell morphology and size**. Morphology of cultured adherent and trypsinized BN rat ASCs **(A,B)** and BM-MSCs **(C,D)**. Scale bar = 100 μm. **(E)** ASC and BM-MSC cell size. Sizes of adherent and trypsinized ASCs (blue bars) and BM-MSCs (red bars). **P < 0.01 vs. ASCs.

Random sampling and flow cytometry analysis confirmed the CD45^−^CD29^+^CD90^+^CD73^+^ phenotype in morphologically uniform cultures of BN rat ASCs and BM-MSCs (Figure 1). Both third-passaged adherent ASCs and BM-MSCs were spindle-shaped and morphologically similar (Figures 2A,C). However, the sizes of attached ASCs and BM-MSCs varied between 100.81 ± 25.14 and 258.06 ± 49.46 μm, respectively (*P* < 0.01). The diameter of trypsinized cells was significantly smaller and differed between 16.6 ± 3.1 μm (ASCs) and 45.5 ± 5.6 μm (BM-MSCs), respectively (*P* < 0.01; Figures 2B,D,E).

### Viability assay

**Figure 3:**
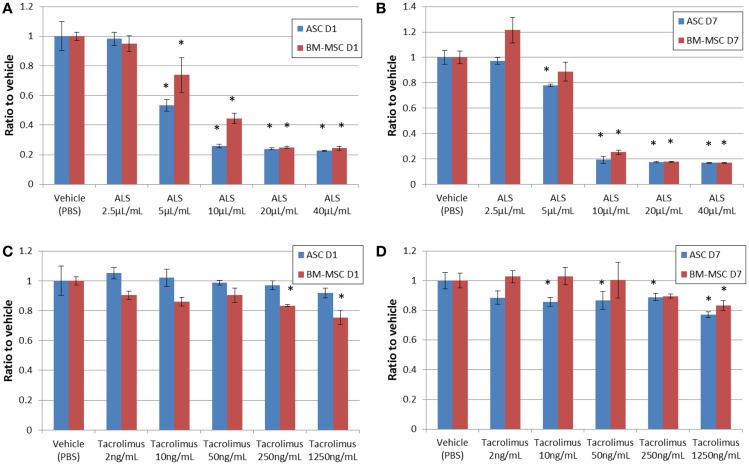
**Cell viability under drug exposure**. MTT assay showing cell viability on day 1 **(A,C)** and day 7 **(B,D)** after adding various concentrations of ALS or tacrolimus. ALS had a suppressive effect on both ASCs and BM-MSCs at higher concentrations from day 1 through day 7 **(A,B)**. Tacrolimus had a suppressive effect on ASCs on day 7 **(C,D)**. *P < 0.05 vs. vehicle group at each time point.

Figure 3 shows cell viability with the MTT assay on day 1 (Figures 3A,C) and day 7 (Figures 3B,D) when cells were exposed to increasing concentrations of ALS. Measurements were taken in triplicates, showing a dose-dependent decrease of cell viability on days 1 and 7 in the presence of ALS. All the values were subtracted from those of the media only group (no cell group). ASCs seemed to be slightly more susceptible to these ALS-related effects compared to BM-MSCs on day 7. Compared to BM-MSCs, ASC viability decreased to 53.92 ± 8.33% and 78.12 ± 4.79% at a concentration of 5 μL/mL at days 1 and 7. The acute effects of tacrolimus on cell viability were not significant in the first 24 h for ASCs and BM-MSCs. However, after incubation with tacrolimus for 7 days, both cell types revealed a dose-dependent sensitivity to the drug. ASCs were comparably more susceptible, with viability decreasing to 88.6 ± 22.91% at 2 ng/mL tacrolimus and 77.26 ± 6.13% at 1250 ng/mL. BM-MSCs reacted to tacrolimus concentrations of 250 and 1,250 ng/mL, (with decreases in cell counts to 97.63 ± 2.08 and 97.20 ± 6.77%), respectively, but not at lower concentrations (Figures 3C,D).

### Proliferation assay

**Figure 4:**
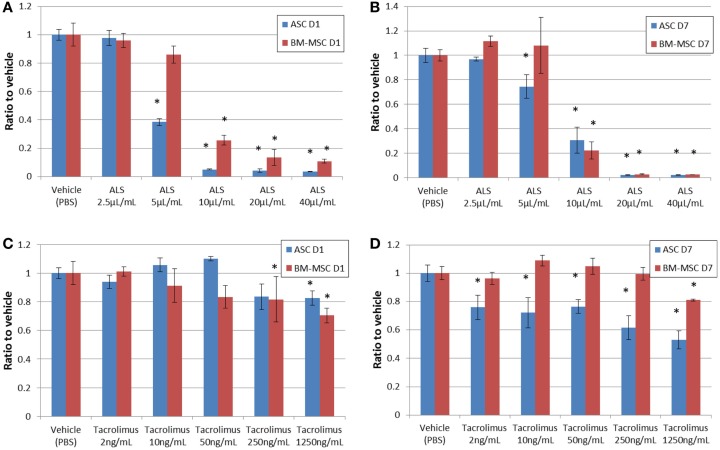
**Cell proliferation under drug exposure**. CyQUANT assay results with a clear dose-dependent effect for ASCs and BM-MSCs under ALS-influence on days 1 **(A)** and 7 **(B)**. CyQUANT assay results after adding tacrolimus on day 1 **(C)** and day 7 **(D)**. CyQUANT assay results are consistent with the MTT assay results. *P < 0.05 vs. vehicle group at each time point.

The CyQUANT assay for cell proliferation showed clear dose-dependent effects for ASCs under the influence of ALS (Figure 4). Cells were exposed to ALS over 24 h (Figure 4A) and 7 days (Figure 4B). All the values were subtracted from those of the media only group (no cell group). At 5 μL/mL ALS, the proliferation of ASCs was markedly reduced at both time points compared to the vehicle group. Increased concentrations of ALS led to an arrest of proliferation. For BM-MSCs, the effect was observed only at concentrations above 10 μL/mL. After the addition of tacrolimus, no marked reaction was detectable within 24 h. After 7 days, ASCs showed a dose-dependent reduction of proliferation rates at very high concentrations, similar to the effect observed for BM-MSCs (Figures 4C,D). The maximal decrease of proliferation was 52.9 ± 3.35% for ASCs and 81.24 ± 4.40% for BM-MSCs, respectively, at 1250 ng/mL. ASCs had higher levels of proliferation on day 1 with gradual reduction in proliferation toward day 7 (Figure 4C), while BM-MSC proliferation improved with tacrolimus incubation reaching higher levels at day 7 (Figure 4D).

### Serum effects on cell viability

**Figure 5:**
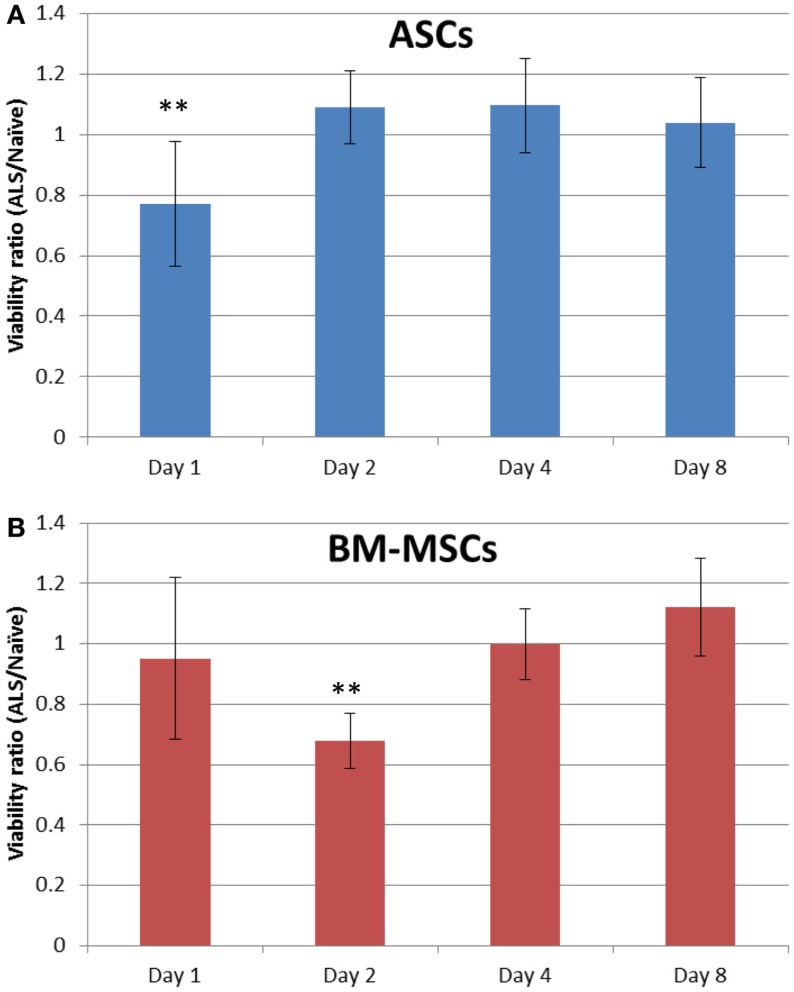
**ASC and BM-MSC susceptibility to ALS**. ASC [blue bars, **(A)**] and BM-MSC [red bars, **(B)**] were incubated for 2 h in 100% serum from ALS-treated or untreated naïve rats. An MTT assay was performed on days 1, 2, 4, and 8. The direct absorbance ratio of cells exposed to serum (as conditioned medium) from ALS-treated and naïve rats is presented. Data are expressed as mean ± SD. **P < 0.01 vs. other days.

Replacing the culture medium of stem cells *in vitro* with serum from ALS-treated rats reduced the viability of ASCs significantly (Figure 5A). On day 1, viability decreased to 71.53 ± 21.27%, which was significant compared to day 2. Tests performed at later time points did not reveal this early detrimental effect. BM-MSCs, however, showed a similar but delayed decrease of viability with significant differences after day 2 (Figure 5B).

### Complement-dependent cytotoxicity assay

**Figure 6:**
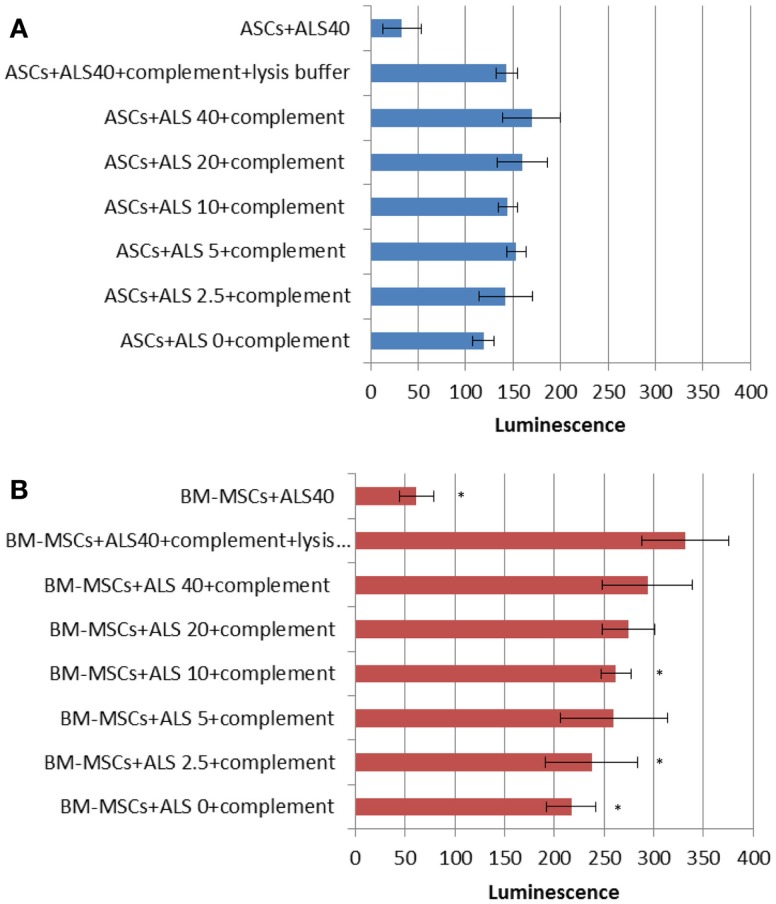
**Complement-dependent cytotoxicity (CDC) under drug exposure**. CDC assay results for ASCs **(A)** and BM-MSCs **(B)**. Higher concentrations of ALS showed cytotoxic effects on BM-MSCs but not ASCs. *P < 0.05 vs. maximal lysis group (cells + ALS40 + complement + lysis buffer).

The CDC assay was utilized to detect cell lysis based on glyceraldehyde-3-phosphate dehydrogenase (GAPDH)-release using rat complement as a probe in the presence of ALS. Whereas the negative control (cells and 40 μL/mL of ALS) did not show a strong response (Figure 6), low concentrations of ALS revealed a CDC with a strong dose-dependency. ASCs did not succumb to these complement-mediated effects to the same extent as BM-MSCs and showed lower levels of luminescence compared to that with BM-MSCs (Figure 6A vs. Figure 6B). Maximal lysis control was obtained by the combination of cells, complement, ALS (40 μL/mL), and lysis buffer.

## Discussion

The success of cell-based immunomodulation depends on facilitating graft acceptance, or ideally tolerance, in the absence of graft-versus-host disease in the field of transplantation. Various drugs and antibodies have been introduced for induction therapy and maintenance-immunosuppression in clinical SOT and have been widely used in clinical VCA protocols (18). All these agents have proven immunosuppressive efficacy, but their collateral bystander effects on specific cell types in the recipient as well on BM-MSCs or ASCs have thus far been less well investigated.

Clinical protocols utilize tolerance induction with lympho-depleting antibodies like ATG or alemtuzumab to suppress the recipient alloresponses. ALS is analogous to ATG and has been evaluated in experimental models of SOT as well as VCA (19). However, the potential detrimental effects of the depletion regimen on MSC viability, proliferation, differentiation, recruitment, and function, as well as on their immunomodulatory potential remain undetermined. Several SOT clinical trials have evaluated BM-MSCs or ASCs as cell therapy strategies in combination with conventional induction/maintenance-immunosuppression and documented the utility and efficacy of such therapies in facilitating reduction of dosing/intensity, frequency, or duration of immunosuppression (20, 21). Perico et al. first demonstrated the effect of ATG on BM-MSCs *in vitro* (20). They exposed BM-MSCs to serum from ATG-treated kidney transplant patients taken postoperatively on days 7 and 14, and found a very low percentage of ATG bound to BM-MSCs compared with ATG bound to peripheral blood mononuclear cells. Franquesa et al. conducted the experiment to test the effect of ATG on ASCs. They tested 0.5–100 ng/mL of ATG, and found that ATG reduced the viability and antiproliferative capacity of ASCs in a dose-dependent manner and converted them into targets for CD8^+^ T cells and natural killer T-cell lysis (22). Polyclonal antibodies such as ATG or ALS are well known induction agents which seemingly are deleterious to stem cell therapies injected concurrently with antibody treatment. The fraction of ASCs or BM-MSCs that remain viable after ALS treatment could proliferate as shown by our results.

Adipose-derived stem cells and BM-MSCs are quite similar in cell surface markers; they are positive for CD29, 73, and 90 and negative for CD45 (Figure 1). Also, they are similar in their proliferative capacity. Studies have shown that primary cultures reach 70–80% confluence in approximately 7–9 days for BM-MSCs and 5–6 days for ASCs (23). In our experiment, both ASCs and BM-MSCs became 80% confluent within 7 days, and were passaged every 7 days. Our results exhibit functional differences for ASCs and BM-MSCs in terms of susceptibility to ALS as well as tacrolimus. The most important finding in our study was that ASCs are more susceptible to the toxicity of ALS and tacrolimus than BM-MSCs (Figures 3B,D and Figures 4B,D). The ALS doses used *in vitro* were calculated to ensure comparability with ALS dosing used in conventional clinical induction protocols. Further, ASCs and BM-MSCs differ in their sensitivity to ALS in peak loss of viability by the timing of exposure to ALS (Figure 5). The influence of tacrolimus on ASC and BM-MSC viability and susceptibility was more pronounced after 7 days of incubation than the immediate effect after 1 day in our study. BM-MSCs demonstrated slightly higher susceptibility to tacrolimus at higher dosages than ASCs. Some of these findings were confirmed in earlier studies with human muscle derived-MSCs (MD-MSCs) exposed to select antibodies or drugs used in SOT. Hoogduijin et al. reported that high systemic tacrolimus levels concentration (100 ng/mL) were detrimental to MD-MSC survival after incubation over one week (24). Pre-incubation of MD-MSCs with tacrolimus, however, augmented their immunosuppressive effect. Interestingly, MD-MSCs inhibited tacrolimus-induced suppression of alloactivated mononuclear cells. Other studies with BM-MSCs demonstrated disparate positive or negative effects of immunosuppressive agents such as tacrolimus, rapamycin, or mycophenolic acid mofetil on human BM-MSCs (25, 26).

In addition, we were able to obtain data on the morphological differences between ASCs and BM-MSCs, especially for trypsinized cells, which are relevant to route of application. In the morphological study, we demonstrated that BM-MSCs (Figures 2B,D,E) were significantly larger in size as compared to ASCs (Figures 2A,C,E). Larger size could make the cells more prone to lung entrapment with reduction of their peripheral homing capacity. This theory is reinforced by recent reports of entrapment of MSCs in the lung capillaries following systemic delivery (27, 28) and may indeed limit clinical impact of BM-MSCs. Various strategies, including peripheral arterial administration (15) or repetitive application to increase the lung bypass have been suggested (27). To our knowledge, this is the first direct morphological comparison of these two MSC types in regards to cell size. Our results show that trypsinized ASCs are only 30% in diameter compared to trypsinized BM-MSCs. In this regard, we speculate that ASCs can more effectively pass the pulmonary capillary bed with less entrapment. Fischer et al. (27) demonstrated that repetitive cell administration would prevent stromal cell loss from the circulation. Repetitive application of ASCs and BM-MSCs has also been reported as a successful strategy for immunomodulation in rat and pig hind-limb transplantation (29, 30).

Our *in vitro* outcomes of the CDC assay advocate for the use of ASCs rather than BM-MSCs in a transplant setting. ASCs seem to be more resistant to the cytotoxic effects mediated through the complement system as tested under the influence of ALS in our study. This may impact stem cell viability or functional efficacy in the early post-transplant phase. To our knowledge, the susceptibility of MSCs has not been tested earlier with regards to the complement system and transplantation.

Our study is the first to characterize the specific effects of two of the most widely used induction and maintenance immunosuppressive agents in SOT (ALS and tacrolimus). Our data offers critical early insights into comparative susceptibility or relative resistance of viability or proliferative capacity of BM-MSCs or ASCs to collateral effects of these agents. These *in vitro* data support the hypothesis that phenotypic, viability, and proliferative characteristics of ASCs or BM-MSCs may positively or negatively impact their homing, survival, and key functional efficacy *in vivo* especially when cell therapy protocols logistically overlap with depletional induction/maintenance regimens. These variables are important to consider in the determination of appropriate timing, dosing, frequency, and cell-type choice in cell therapy protocols used adjunctively with conventional immunosuppressive induction or maintenance regimens. We are currently exploring *in vivo* translation of these findings in experimental limb VCA models.

## Conflict of Interest Statement

The authors declare that the research was conducted in the absence of any commercial or financial relationships that could be construed as a potential conflict of interest.

## Abbreviations

ALS, anti-lymphocyte serum; ASCs, adipose-derived stem cells; ATG, anti-thymocyte globulin; BM-MSCs, bone marrow-derived mesenchymal stem cells; BN rats, Brown Norway rats; CDC, complement-dependent cytotoxicity; MSCs, mesenchymal stem cells; SOT, solid organ transplantation; VCA, vascularized composite tissue allotransplantation.
